# L-carnitine protects against methotrexate-induced hepatotoxicity via regulation of apoptosis and metabolic pathways

**DOI:** 10.1007/s00210-026-05196-x

**Published:** 2026-03-24

**Authors:** Sakir Pekgoz, Zafer Usta, Yagmur Kara, Ozlem Ozmen

**Affiliations:** 1 Department of Medical Services and Techniques, Isparta Health Services Vocational School, Süleyman Demirel University, 32260 Isparta, Turkey; 2 Department of Genetics, Faculty of Veterinary Medicine, Burdur Mehmet Akif Ersoy University, 15030 Burdur, Turkey; 3 Department of Internal Medicine, Emergency Service, Isparta City Hospital, 32200 Isparta, Turkey; 4 Department of Pathology, Faculty of Veterinary Medicine, Burdur Mehmet Akif Ersoy University, 15030 Burdur, Turkey

**Keywords:** Methotrexate, L-carnitine, Hepatotoxicity, Oxidative stress, Apoptosis, CD38, GLUT1, BAX/BCL2

## Abstract

Methotrexate (MTX) is a widely used chemotherapeutic and immunosuppressive drug; however, its clinical application is limited by dose-dependent hepatotoxicity mediated by oxidative stress, mitochondrial dysfunction, and apoptosis. This study aimed to investigate the potential hepatoprotective effects of L-carnitine (LCR), a mitochondrial cofactor with antioxidant and anti-apoptotic properties, in a rat model of MTX-induced hepatotoxicity. Twenty-four male Wistar rats were randomly divided into four groups: Control, MTX (20 mg/kg, i.p., single dose), LCR (100 mg/kg/day, oral) and MTX + LCR. After 10 days, liver tissues were examined histopathologically, immunohistochemically for CD38 and GLUT1, and by qPCR for BAX and BCL2 expression. Serum alanine aminotransferase (ALT) and aspartate aminotransferase (AST) levels were also measured. MTX administration induced histological injury, upregulated CD38 and GLUT1 expression and altered apoptosis-related genes (BAX↑, BCL2↓) with a trend toward elevated ALT and AST levels. LCR co-treatment improved hepatic histoarchitecture, reduced CD38 and GLUT1 expression and partially normalized BAX/BCL2 expression, with a tendency to restore serum enzyme activity. L-carnitine may attenuate MTX-induced hepatotoxicity by modulating oxidative stress, metabolic stress and apoptotic signaling. These findings suggest a potential role for L-carnitine as an adjuvant to improve hepatic safety during MTX therapy.

## Introduction

Methotrexate (MTX) is a folate antagonist widely used for its immunosuppressive and antiproliferative properties in the treatment of malignancies, autoimmune disorders such as rheumatoid arthritis and various dermatological conditions (Cronstein and Aune 2020). Despite its broad clinical applicability, MTX is often associated with dose-dependent organ toxicity, particularly hepatotoxicity, which significantly limits its therapeutic potential. MTX-induced liver damage is a well-documented adverse effect and remains a major concern in long-term therapy. This toxicity primarily stems from oxidative stress, mitochondrial dysfunction, and apoptosis, all of which disrupt hepatic homeostasis (Ezhilarasan 2021; Schmidt et al. 2022).

The liver, as a central organ in metabolism and detoxification, is particularly vulnerable to xenobiotic-induced oxidative damage. MTX administration has been shown to provoke an increase in reactive oxygen species (ROS), lipid peroxidation and mitochondrial impairment, resulting in hepatocellular degeneration, necrosis and inflammatory infiltration (Schmidt et al. 2022; El-Sheikh et al. 2015). Furthermore, MTX activates both extrinsic and intrinsic apoptotic pathways, as evidenced by increased expression of pro-apoptotic markers such as BAX and decreased levels of anti-apoptotic proteins like BCL2 (Sener et al. 2006; Schmidt et al. 2022). Apoptotic imbalance contributes to irreversible liver damage and functional decline, making it essential to explore protective agents that can mitigate these molecular alterations.

Routine liver function tests, including serum alanine aminotransferase (ALT) and aspartate aminotransferase (AST), serve as key clinical indicators of hepatocellular injury (Tousson et al., 2014). However, these biochemical indices are insufficient to capture the complexity of intracellular stress responses. For this reason, a more detailed molecular investigation is often required. In this context, glucose transporter 1 (GLUT1) and CD38 have gained attention as surrogate markers of hepatic oxidative and metabolic stress. GLUT1 facilitates the basal uptake of glucose, which is vital for energy metabolism, particularly under pathological conditions. An upregulation of GLUT1 expression reflects an adaptive response to increased cellular demand for ATP and antioxidant defense (Li et al. 2015). CD38, a multifunctional ectoenzyme involved in NAD⁺ metabolism and calcium signaling, is closely linked to mitochondrial function, redox balance, and inflammatory signaling cascades (Flanagan et al. 2010). Aberrant activation of CD38 has been implicated in promoting oxidative damage, hepatocellular senescence, and immune dysregulation in MTX-induced toxicity models (Li et al. 2015; Schmidt et al. 2022).

L-carnitine (LCR) is a naturally occurring zwitterionic molecule that plays a pivotal role in the transport of long-chain fatty acids into the mitochondrial matrix for β-oxidation and subsequent ATP generation (Bremer 1983; Steiber et al. 2004). Beyond its well-established role in energy metabolism, LCR exhibits potent antioxidant, anti-inflammatory and anti-apoptotic properties. These effects are largely attributed to its capacity to stabilize mitochondrial membranes, scavenge free radicals and modulate intracellular signaling pathways involved in cell survival (Sener et al. 2006; Flanagan et al. 2010). In various models of drug-induced organ injury, including nephrotoxicity and cardiotoxicity, L-carnitine has demonstrated significant protective efficacy. Of particular relevance, previous studies have shown that LCR supplementation reduces lipid peroxidation, restores endogenous antioxidant levels, and inhibits caspase activation in MTX-induced hepatic injury (Sener et al. 2006; Khatab et al. 2022).

Moreover, the therapeutic benefits of L-carnitine in the setting of MTX toxicity are supported by findings indicating its role in regulating BAX/BCL2 expression. Radwan et al. (2023) reported that LCR co-administration restored the apoptotic balance and improved liver architecture in rats treated with MTX, possibly via modulation of Notch1/Hes-1 signaling pathways. Khatab et al. (2022) further demonstrated that LCR attenuated hepatocyte apoptosis in isolated hepatocytes, supporting its direct cytoprotective role at the cellular level. These studies, together with histological and biochemical analyses, provide a compelling rationale for the continued evaluation of LCR as an adjuvant therapy to mitigate MTX-associated hepatotoxicity.

Despite its promising hepatoprotective potential, the mechanisms by which L-carnitine exerts its protective effects in MTX-induced liver injury remain incompletely understood. A key question is whether LCR can simultaneously modulate oxidative stress, mitochondrial dysfunction, metabolic stress markers (GLUT1, CD38) and apoptotic signaling pathways (BAX/BCL2) in a coordinated fashion. To date, very few studies have investigated this question using a comprehensive multimodal approach.

Therefore, the present study aimed to investigate the potential protective effects of L-carnitine against MTX-induced hepatotoxicity in a rat model using a multifaceted experimental design. Specifically, we sought to evaluate the impact of LCR on serum liver enzymes (ALT, AST), histopathological architecture, immunohistochemical expression of GLUT1 and CD38, and gene expression of BAX and BCL2. By correlating histological, biochemical, immunological, and molecular data, this study provides an integrated analysis of how L-carnitine may ameliorate hepatic injury induced by MTX. Our findings contribute to a growing body of literature supporting the use of L-carnitine as a therapeutic adjuvant in chemotherapeutic regimens, especially in patients at risk for drug-induced hepatotoxicity.

## Objectives

This study aimed to investigate the potential hepatoprotective effects of L-carnitine (LCR) against methotrexate (MTX)-induced liver toxicity in a rat model. A comprehensive, multimodal approach was employed, integrating serum biochemical markers (ALT, AST), histopathological evaluation, immunohistochemical staining (GLUT1, CD38) and quantitative gene expression analysis (BAX and BCL2). The primary objective was to determine whether LCR could alleviate MTX-induced oxidative stress, inflammation and apoptosis, thereby preserving hepatic structure and function.

## Hypotheses

It was hypothesized that:

MTX administration will induce significant hepatotoxicity, characterized by:

- Increased liver injury scores in histopathological examinations (necrosis, inflammation, and hepatocellular degeneration),
- Elevated expression of oxidative stress and metabolic markers (CD38 and GLUT1) in immunohistochemical analyses,
- Upregulation of pro-apoptotic BAX and downregulation of anti-apoptotic BCL2 mRNA levels, reflecting enhanced apoptotic signaling.

L-carnitine (LCR) co-treatment will ameliorate MTX-induced hepatic injury, demonstrated by:

- Improved histological architecture, with decreased necrotic and inflammatory lesions,
- Reduced expression of CD38 and GLUT1, indicating attenuation of oxidative stress,
- Suppression of BAX and restoration of BCL2 expression, suggesting inhibition of apoptosis.

Mechanistic hypothesis:

The hepatoprotective effects of LCR are mediated through modulation of oxidative stress and apoptotic pathways, highlighting its potential as a therapeutic adjuvant in MTX-based treatments.

## Materials and methods

### Animals and ethical approval

A total of 24 healthy male Wistar albino rats (weighing 250–300 g, 8–10 weeks old) were used in this study, with six animals allocated to each experimental group (n = 6). The sample size was determined based on previous MTX-induced hepatotoxicity studies reporting moderate-to-strong effect sizes and an a priori power analysis (β = 0.80, α = 0.05) performed using G*Power software (Faul et al. 2007). This sample size was considered adequate to detect biologically and statistically significant differences while minimizing animal use in line with the 3Rs principle (Replacement, Reduction, and Refinement) (Russell and Burch 1959; National Research Council, 2011).

Animals were acclimatized for one week before the experiments and maintained under standard laboratory conditions (22 ± 2 °C, 55 ± 5% relative humidity, 12-h light/12-h dark cycle) with ad libitum access to standard pellet chow and water. All experimental procedures were approved by the Burdur Mehmet Akif Ersoy University Animal Experiments Local Ethics Committee (Approval No: 1379, dated 02.10.2024) and conducted according to the ARRIVE guidelines (Percie du Sert et al. 2020).

### Experimental groups

The rats were randomly divided into four experimental groups (n = 6 per group):

- **Control group**: Received physiological saline (0.9% NaCl, 1 mL, i.p.) once daily for 10 consecutive days.
- **MTX group**: Received physiological saline (0.9% NaCl, 1 mL, i.p.) once daily for 10 days, with a single dose of methotrexate (20 mg/kg, i.p.; Methotrexate DBL, 500 mg/20 mL vial, Koçak Farma, Turkey) administered on day 5 (Samdanci et al. 2019).
- **MTX + LCR group**: Received L-carnitine (200 mg/kg, i.p., 1 mL) once daily for 10 days, with a single dose of methotrexate (20 mg/kg, i.p.) administered on day 5 (Sezen et al. 2008).
- **LCR group**: Received L-carnitine (200 mg/kg, i.p., 1 mL) once daily for 10 days, with a single dose of physiological saline (0.9% NaCl, 1 mL, i.p.) administered on day 5 (Sezen et al. 2008).

Ketamine hydrochloride (Keta-Control) and xylazine (Control %10) were supplied by Doğa İlaç (İstanbul, Türkiye). All other reagents were of analytical grade. On day 11, animals were anesthetized with ketamine (90 mg/kg) and xylazine (10 mg/kg), and euthanized for tissue collection.

This experimental design allowed evaluation of the protective effects of LCR administered after methotrexate-induced hepatotoxicity.

### Histopathological evaluation

Liver tissues fixed in 10% neutral-buffered formalin were subjected to routine histological processing, embedded in paraffin, and sectioned at 4–5 μm using a rotary microtome. Sections were mounted on poly-L-lysine–coated slides, deparaffinized in xylene, rehydrated through a graded ethanol series, and stained with hematoxylin and eosin (H&E).

The sections were evaluated under a light microscope (Leica DM750, Germany) by a pathologist blinded to group allocation. Histopathological changes were semi-quantitatively scored using a four-point grading system (0–3) for the following criteria:

- Hepatocyte degeneration and ballooning
- Sinusoidal congestion
- Focal or diffuse necrosis
- Inflammatory cell infiltration (lymphocytes, neutrophils)
- Cytoplasmic vacuolization
- Hemosiderin deposition and hemorrhage

The total hepatic injury score was calculated by summing all parameters for each animal (Table 1).

**Table 1:** Histopathology score of hepatic lesions

Histological criteria	Severity	Description	Score
Hemorrhage	Absent		0
	Mild	< 3 foci	1
	Marked	4–6 foci	2
	Severe	> 7 foci	3
Inflammation	None		0
	Moderate	Scattered	1
	Marked	Foci	2
	Severe	Diffuse	3
Necrosis	Absent	0%	0
	Mild	< 10%	1
	Marked	10–50%	2
	Severe	> 50%	3

### Immunohistochemical analysis (CD38 and GLUT1)

Paraffin-embedded liver Sects. (4–5 µm) were mounted on poly-L-lysine–coated slides and incubated at 60 °C overnight. Following deparaffinization in xylene and rehydration through graded ethanol series, endogenous peroxidase activity was quenched with 3% H₂O₂ in methanol for 10 min. Antigen retrieval was performed in citrate buffer (pH 6.0) using a microwave oven for 15 min. Non-specific binding was blocked with normal goat serum for 20 min at room temperature.

Sections were incubated overnight at 4 °C with the following primary antibodies: anti-GLUT1 (rabbit polyclonal, 1:200, Abcam, UK) and anti-CD38 (rabbit monoclonal, 1:100, Abcam, UK). The next day, sections were treated with biotinylated secondary antibodies and streptavidin–HRP (Thermo Scientific, USA). Immunoreactivity was visualized using 3,3′-diaminobenzidine (DAB) as chromogen and counterstained with Mayer’s hematoxylin.

For each sample, ten randomly selected high-power fields (400 × magnification) were evaluated using a light microscope (Leica DM750, Germany) by a pathologist blinded to the group allocations. Staining intensity was scored semi-quantitatively as: 0 = negative, 1 = weak, 2 = moderate, 3 = strong. Quantitative results (mean ± SE) for the percentage of positive hepatocytes are provided in Fig. 5.

### Quantitative real-time PCR (RT-qPCR) analysis

Total RNA was extracted from ~ 50 mg frozen liver tissue using the RNeasy Plus Mini Kit (Qiagen, Germany). RNA purity and concentration were determined spectrophotometrically (NanoDrop ND-1000). Only samples with A260/A280 = 1.8–2.1 were used.

cDNA was synthesized using the High-Capacity cDNA Reverse Transcription Kit (Thermo Fisher Scientific). RT-qPCR was performed on a Bio-Rad CFX96 Real-Time PCR System using SYBR Green Master Mix. GAPDH was used as the housekeeping gene. Primer sequences are listed in Table 2.

**Table 2:** Primer sequences and expected product sizes for target and reference genes

Gene	Primer Sequence (5’–3’)	Product Size
BAX	F: GCAGAGGATGATTGCTGATGT R: CCTTGAGCACCAGTTTGCTA	143 bp
BCL2	F: CATCTCATGCCAAGGGGGAA R: TATCCCACTCGTAGCCCCTC	284 bp
GAPDH (housekeeping)	F: CAAGGTCATCCCAGAGCTGAA R: CATGTAGGCCATGAGGTCCAC	340 bp

BAX: Bcl-2 associated X protein; BCL2: B-cell lymphoma 2; GAPDH: Glyceraldehyde-3-phosphate dehydrogenase

Relative gene expression levels were calculated using the 2^ − ΔΔCt method (Livak and Schmittgen 2001).

### Serum biochemical analysis (ALT and AST)

At the end of the experimental period, blood samples were collected via cardiac puncture under anesthesia. Serum was separated by centrifugation at 3000 rpm for 10 min using a refrigerated centrifuge (Eppendorf 5415 R, Germany) and stored at − 80 °C in a deep freezer (WiseCryo WUF 400, Korea) until analysis. Serum alanine aminotransferase (ALT) and aspartate aminotransferase (AST) activities were measured in duplicate using an automated biochemical analyzer (Beckman Coulter AU 5800, Japan) according to the manufacturer’s instructions. Results were expressed in units per liter (U/L).

### Statistical analysis

Data were expressed as mean ± standard deviation (SD). Statistical analysis was performed using IBM SPSS Statistics for Windows, Version 25.0 (IBM Corp., Armonk, NY, USA). Normality was tested using the Shapiro–Wilk test. Group comparisons were performed using one-way ANOVA, followed by Tukey’s post hoc test. Statistical significance was considered at *p* ≤ 0.05.

Significance levels were denoted as follows:

- *p* ≤ 0.05 (*),
- *p* ≤ 0.01 (**),
- *p* ≤ 0.001 (***),
- ns: not significant.

## Results

### General observations

No mortality occurred in any experimental group throughout the study. Rats in the MTX group exhibited reduced food intake, mild lethargy, and ruffled fur after MTX administration, consistent with acute hepatotoxicity models. Rats in the LCR and MTX + LCR groups remained generally active, indicating a partial protective effect of L-carnitine.

### Histopathological findings

**Fig. 1:**
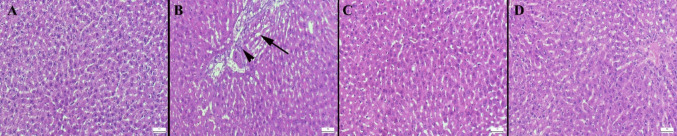
Representative photomicrographs of liver sections (H&E, scale bar = 20 µm). (**A**) Control group showing normal hepatic histology. (**B**) MTX group displaying hepatocellular necrosis, vascular congestion (arrow), and inflammatory cell infiltration (arrowhead). (**C**) LCR group with preserved architecture and no pathological changes. (**D**) MTX + LCR group showing improved tissue integrity with minimal lesions

Histopathological examination of liver sections from the Control (Fig. 1A) and LCR-only (Fig. 1C) groups revealed well-preserved hepatic architecture, characterized by normal hepatocyte morphology, intact central veins, and clearly defined hepatic cords and sinusoids. No pathological alterations were observed in these groups.

**Fig. 2:**
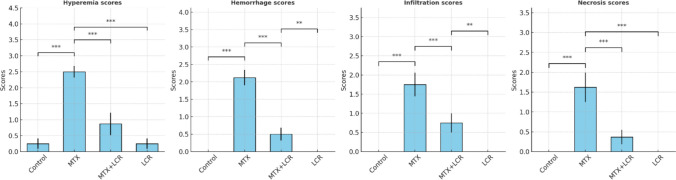
Histopathological scoring of liver tissue. Bars represent mean ± SEM for Control, MTX, MTX + LCR and LCR groups. MTX administration significantly increased hyperemia, hemorrhage, infiltration and necrosis compared to control. L-carnitine co-treatment (MTX + LCR) partially attenuated these changes. Statistical significance is indicated as * *p* < 0.05, ** *p* < 0.01, *** *p* < 0.001

In contrast, the MTX-treated group (Fig. 1B) exhibited pronounced histological damage, predominantly localized in the centrilobular region surrounding the central veins. The lesions included moderate hyperemia (vascular congestion), extensive hepatocellular necrosis, marked inflammatory cell infiltration, and mild sinusoidal and parenchymal hemorrhage (Fig. 2).

**Fig. 3:**
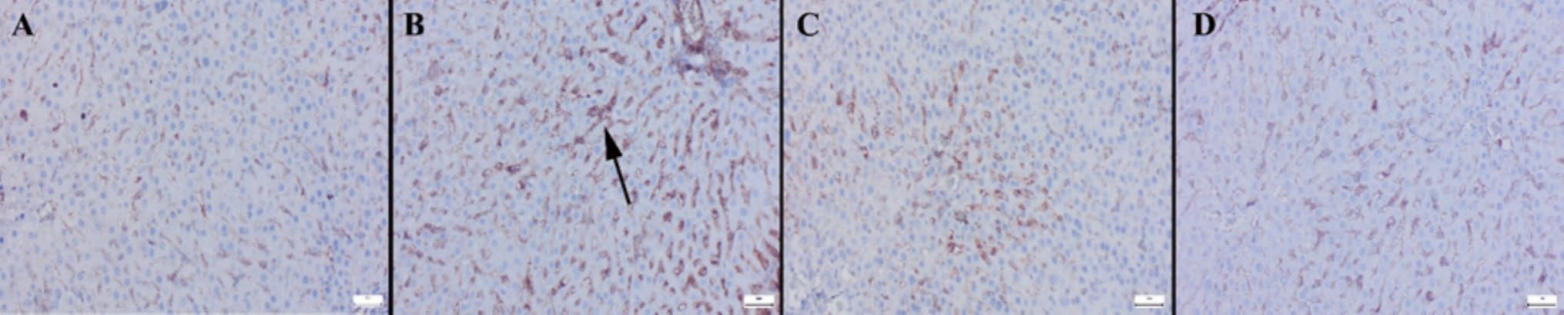
Representative immunohistochemical staining of CD38 in liver tissue sections from different experimental groups (streptavidin–biotin peroxidase method; scale bars = 50 µm). (**A**) Control group: Exhibits negative to minimal CD38 immunoreactivity, reflecting baseline expression under physiological conditions. (**B**) MTX group: Shows a significant upregulation of CD38, characterized by intense cytoplasmic and membranous staining predominantly localized in hepatocytes and Kupffer cells (indicated by black arrows), indicative of enhanced inflammatory and immune activation following MTX-induced hepatic injury. (**C**) MTX + LCR group: Demonstrates markedly reduced CD38 expression relative to the MTX group, supporting the anti-inflammatory and hepatoprotective properties of LCR co-treatment. (**D**) LCR group: Displays minimal to absent CD38 staining comparable to the control group, confirming that LCR administration alone does not provoke CD38 overexpression or hepatic immune activation

**Fig. 4:**
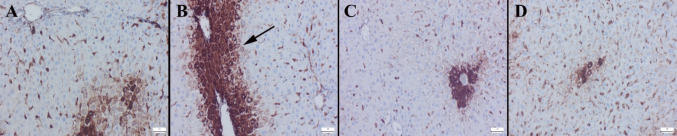
Representative immunohistochemical staining of GLUT1 in liver tissue sections across experimental groups (streptavidin–biotin peroxidase method; scale bars = 50 µm). (**A**) Control group: Exhibits slight GLUT1 immunoreactivity, localized to scattered hepatocytes, consistent with normal physiological glucose transport in hepatic tissue. (**B**) MTX group: Demonstrates a pronounced increase in GLUT1 expression, with intense cytoplasmic staining mainly observed in hepatocytes and Kupffer cells (black arrows), indicative of elevated metabolic and oxidative stress triggered by MTX-induced hepatic injury. (**C**) MTX + LCR group: Shows a significant reduction in GLUT1 immunoreactivity compared to the MTX group, suggesting that LCR treatment effectively mitigates MTX-associated metabolic disturbances in the liver. (**D**) LCR group: Displays minimal to absent GLUT1 staining, comparable to the control group, indicating that LCR administration alone does not significantly affect GLUT1 expression under normal conditions

Notably, liver tissue from the MTX + LCR co-treatment group (Fig. 1D) demonstrated substantial preservation of hepatic architecture compared to the MTX group. Hepatocellular necrosis, inflammatory infiltration, and vascular congestion were markedly reduced, and the overall tissue morphology closely resembled that of the control animals, with only minimal histopathological alterations (Figs. 3 and 4).

### Immunohistochemical findings (GLUT1 and CD38 Expression)

**Fig. 5:**
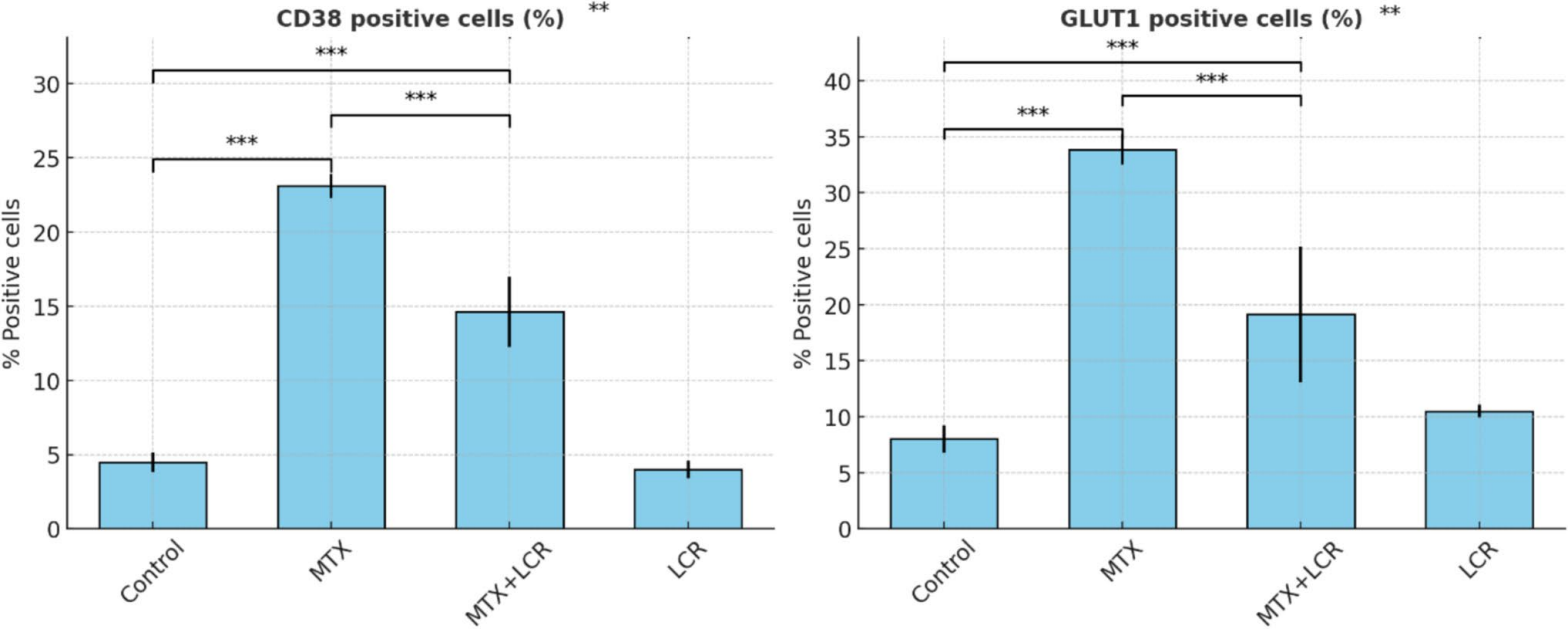
Immunohistochemical analysis of hepatic CD38 and GLUT1 expression. Bars represent mean ± SEM of positive cell percentages in liver tissue from Control, MTX, MTX + LCR and LCR groups. MTX treatment significantly increased CD38 and GLUT1 positive cells compared to control, while L-carnitine co-treatment (MTX + LCR) attenuated these elevations. Statistical significance is indicated as * *p* < 0.05, ** *p* < 0.01, *** *p* < 0.001

Immunohistochemical analysis revealed minimal expression of both CD38 and GLUT1 in the Control and LCR-only groups, consistent with normal physiological conditions and a low basal inflammatory state in hepatic tissues. Staining was weak and predominantly localized to scattered hepatocytes, with negligible immunoreactivity detected in non-parenchymal cells. Quantitative image analysis confirmed low expression levels in these groups (Control: CD38 = 4.50 ± 0.68%; GLUT1 = 8.00 ± 1.22%; LCR-only: p > 0.05 vs. Control) (Fig. 5).

In stark contrast, MTX administration induced a pronounced upregulation of both markers, with significantly higher percentages of CD38-positive hepatocytes (23.12 ± 0.81%) and GLUT1-positive hepatocytes (33.87 ± 1.35%) compared to Control (*p* < 0.001). Immunostaining was diffusely distributed throughout the hepatic parenchyma, with intense cytoplasmic and membranous localization in hepatocytes, alongside moderate expression in inflammatory infiltrates and bile duct epithelial cells reflecting MTX-induced oxidative and metabolic stress.

Co-treatment with LCR significantly reduced CD38 (14.62 ± 2.38%) and GLUT1 (19.12 ± 6.05%) expression compared to MTX alone (*p* < 0.001 for both), although values remained higher than Control (*p* < 0.05). This attenuation was accompanied by visibly weaker staining intensity and reduced distribution across hepatic tissue, indicating that LCR mitigates MTX-induced oxidative stress and inflammatory damage (Fig. 5).

Representative micrographs illustrating the distribution and intensity differences of GLUT1 and CD38 staining across groups are shown in Figs. 3 and 4.

Collectively, these immunohistochemical results provide strong evidence that LCR confers significant hepatoprotection against methotrexate-induced liver injury through modulation of inflammatory pathways, reduction of oxidative stress, and regulation of glucose transporter expression.

### Gene expression analysis (BAX and BCL2 mRNA levels)

RT-qPCR analysis of hepatic BAX and BCL2 mRNA expression, normalized to GAPDH, revealed significant alterations among the experimental groups. All reactions produced single, sharp peaks in the melt curve analysis, confirming primer specificity, with amplification efficiencies ranging between 92 and 105%. Each sample was run in triplicate, and the mean Cq values were used for relative quantification by the 2^-ΔΔCt method.

In the Control and LCR-only groups, BAX expression levels were low and BCL2 expression was comparatively high, reflecting normal anti-apoptotic balance. MTX treatment markedly upregulated BAX mRNA (log₂ fold-change =  + 1.31) and downregulated BCL2 mRNA (log₂ fold-change = –0.99) compared to Control, indicating activation of pro-apoptotic signaling.

**Fig. 6:**
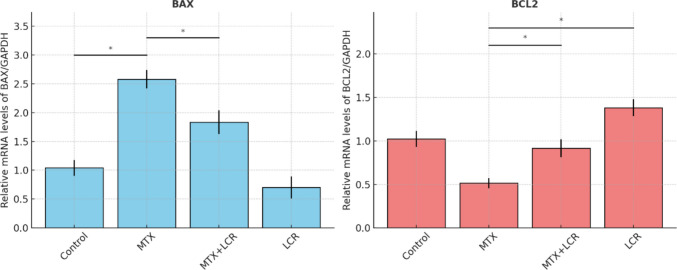
Relative mRNA expression of pro-apoptotic (BAX) and anti-apoptotic (BCL2) genes in liver tissue. Bars represent mean ± SEM of BAX and BCL2 mRNA levels normalized to GAPDH in Control, MTX, MTX + LCR, and LCR groups. MTX administration significantly upregulated BAX and downregulated BCL2 compared to the control group, while L-carnitine co-treatment (MTX + LCR) partially restored their expression toward normal levels. Statistical significance is indicated as * *p* < 0.05

Co-treatment with LCR significantly attenuated these changes: BAX expression was reduced (log₂ fold-change =  + 0.94; 95% CI: + 0.51 to + 1.37; p < 0.001 vs. MTX) and BCL2 expression increased (log₂ fold-change = –0.58; 95% CI: –0.96 to –0.20; *p* < 0.01 vs. MTX). However, BAX remained slightly higher and BCL2 slightly lower than in Control (p < 0.05), suggesting partial but not complete normalization (Fig. 6).

These transcriptional trends align with the histopathological and immunohistochemical findings, supporting the hypothesis that LCR exerts anti-apoptotic effects in MTX-induced hepatotoxicity by modulating BAX/BCL2 balance.

### Serum biochemical analysis (ALT and AST levels)

**Fig. 7:**
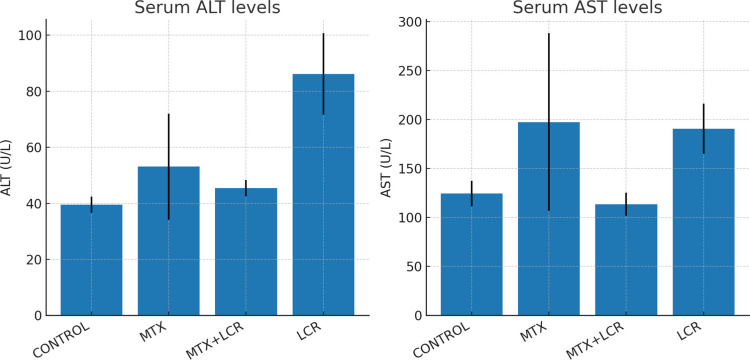
Serum alanine aminotransferase (ALT) and aspartate aminotransferase (AST) levels across experimental groups. Bars represent mean ± SEM values. No statistically significant differences were observed among groups (one-way ANOVA, ALT: F(3,21) = 2.51, p = 0.087; AST: F(3,21) = 0.67, p = 0.581). L-carnitine co-treatment (MTX + LCR) showed a tendency to attenuate MTX-induced elevations in ALT and partially normalize AST levels compared to the MTX group

As shown in Fig. 7, serum alanine aminotransferase (ALT) and aspartate aminotransferase (AST) activities varied across experimental groups.

For ALT, the MTX group exhibited slightly lower mean levels compared to the Control group, whereas the LCR group displayed the highest values, with one prominent outlier (165.4 U/L). Co-treatment with LCR (MTX + LCR) resulted in ALT levels comparable to the Control group, suggesting partial normalization. Statistical analysis revealed no significant group differences (one-way ANOVA: F(3,21) = 2.51, p = 0.087), although a visible trend toward reduction in ALT levels was observed in the MTX + LCR group compared to MTX alone.

For AST, the LCR group also showed the highest values, again with a marked outlier (740.4 U/L). MTX administration did not markedly increase AST compared to Control, while MTX + LCR co-treatment yielded values closer to the Control range. ANOVA results indicated no significant group differences (F(3,21) = 0.67, p = 0.581), yet the distribution pattern suggested that L-carnitine co-administration mitigated extreme AST elevations.

Overall, despite the lack of statistical significance, the graphical distribution patterns suggest that L-carnitine may attenuate MTX-associated alterations in liver enzyme activities.

## Discussion

In this study, we evaluated the potential hepatoprotective effects of L-carnitine (LCR) in a rat model of methotrexate (MTX)-induced liver injury. Our multimodal approach including biochemical, histological, immunohistochemical and gene expression analyses allowed for a comprehensive understanding of the molecular and cellular mechanisms underlying MTX toxicity and LCR-mediated protection. The findings revealed that MTX administration resulted in liver damage, characterized by elevated transaminase levels, tissue necrosis, metabolic dysregulation (GLUT1, CD38) and apoptotic activation (BAX↑, BCL2↓). Importantly, co-treatment with LCR mitigated these adverse effects, suggesting that LCR acts through multiple complementary mechanisms to preserve hepatic structure and function.

Below, we discuss these findings in the context of previous literature, structured around key functional domains: biochemical indicators, histological architecture, oxidative/metabolic stress, apoptotic signaling and mechanistic perspectives.

### Novelty and context

To the best of our knowledge, this is the first study to employ immunohistochemical CD38 and GLUT1 as hepatic oxidative/metabolic stress readouts specifically in an MTX-induced hepatotoxicity model. Prior work has detailed oxidative/mitochondrial stress and apoptosis in MTX liver injury (Ezhilarasan 2021; Schmidt et al. 2022). Positioning CD38/GLUT1 alongside BAX/BCL2 and blinded histopathology provides an integrated, tissue-level perspective directly anchored to our measured endpoints.

### Biochemical markers indicate partial hepatoprotection

In this acute model, ALT/AST group means did not separate significantly, despite clear tissue-level and molecular injury signals. This is consistent with known limitations of aminotransferases particularly over short windows or focal/resolving injury where serum enzymes may under-reflect histological damage (McGill 2016). Even so, the MTX + LCR values trended toward control, which is biologically coherent with LCR-mediated protection. Across MTX models, representative antioxidant/anti-inflammatory interventions show ALT/AST attenuation with improved histology (Behairy et al. 2024; Chauhan et al. 2020; AbdelKader et al. 2023; Alfwuaires 2022; Morsy et al. 2022). Within the L-carnitine literature, MTX studies similarly document hepatoprotection and anti-apoptotic effects (Sener et al. 2006; Khatab et al. 2022; Radwan et al. 2023). Taken together, the lack of statistical separation in ALT/AST here does not contradict hepatoprotection; rather, it underscores the value of convergent tissue-level readouts when interpreting biochemical markers in MTX injury.

### Histopathological improvements support tissue-level protection

MTX produced the expected centrilobular necrosis, inflammatory infiltration, vascular congestion and focal hemorrhage, yielding higher blinded injury scores (Figs. 1–2) and aligning with its canonical hepatotoxic profile (Ezhilarasan 2021). L-carnitine preserved lobular architecture and reduced necrosis/inflammation, lowering composite lesion scores. These findings agree with prior MTX reports of LCR-mediated histological protection (Sener et al. 2006; Khatab et al. 2022; Radwan et al. 2023) and mirror improvements seen with other antioxidant/anti-inflammatory strategies (Behairy et al. 2024; AbdelKader et al. 2023; Alfwuaires 2022; Morsy et al. 2022; Dar et al. 2021; Tunali-Akbay et al. 2010; Dalaklioglu et al. 2013).

### L-carnitine regulates metabolic stress markers (CD38 and GLUT1)

MTX markedly increased CD38 and GLUT1 immunopositivity; LCR significantly reduced both (partial normalization). To our knowledge, this is the first MTX hepatotoxicity study to deploy IHC-based CD38/GLUT1 as hepatic stress readouts. Mechanistically, the pattern is compatible with L-carnitine’s canonical roles in mitochondrial fatty-acid transport/energy support and membrane stabilization (Bremer 1983; Steiber et al. 2004). Although most MTX studies did not assay CD38/GLUT1 directly, agents that blunt oxidative/inflammatory stress (AbdelKader et al. 2023; Alfwuaires 2022; Morsy et al. 2022; Hussein et al. 2020; Dar et al. 2021; Abdelaziz et al. 2020; Behairy et al. 2024) consistently improve tissue injury, supporting the biological plausibility of the CD38/GLUT1 decrease we observe with LCR. Moreover, the observed reduction in GLUT1 expression following L-carnitine administration may extend beyond a simple modulation of glucose transport. GLUT1 upregulation is often associated with impaired mitochondrial respiration and a compensatory shift toward glycolysis under hypoxic or stress conditions. Previous studies have demonstrated that L-carnitine supports mitochondrial oxidative phosphorylation and improves oxygen utilization, thereby mitigating pseudo-hypoxic signaling observed in tumor microenvironments (Airley et al. 2001; Cretenet et al. 2016). Thus, the downregulation of GLUT1 in our model may reflect enhanced mitochondrial efficiency and reduced metabolic stress, reinforcing the interpretation of L-carnitine as a bioenergetic stabilizer.

### Apoptosis modulation through BAX and BCL2 expression

MTX shifted the apoptotic axis toward cell death (BAX↑/BCL2↓), consistent with mitochondrial-pathway activation. L-carnitine partially re-balanced this axis (BAX↓/BCL2↑) in parallel with histological improvement. Similar anti-apoptotic effects are reported in MTX models (Sener et al. 2006; Khatab et al. 2022; Radwan et al. 2023) and with other interventions that reduce oxidative/inflammatory stress (AbdelKader et al. 2023; Alfwuaires 2022; Morsy et al. 2022; Hussein et al. 2020; Dar et al. 2021; Abdelaziz et al. 2020; Kızıl et al. 2023).

### Multimodal protection by L-carnitine

Across independent endpoints, LCR attenuated tissue-level metabolic/oxidative stress (CD38/GLUT1↓), re-balanced apoptosis (BAX↓/BCL2↑) and improved histopathology, with ALT/AST trending toward control. This multimodal pattern accords with L-carnitine’s bioenergetic/cytoprotective profile and echoes prior MTX reports (Sener et al. 2006; Khatab et al. 2022; Radwan et al. 2023). From a translational perspective, these data motivate exploration of combination regimens (e.g., with thymoquinone or resveratrol) and evaluation in chronic/cumulative MTX paradigms where metabolic and apoptotic stress are sustained.

### Mechanistic overview supported by measured endpoints

**Fig. 8:**
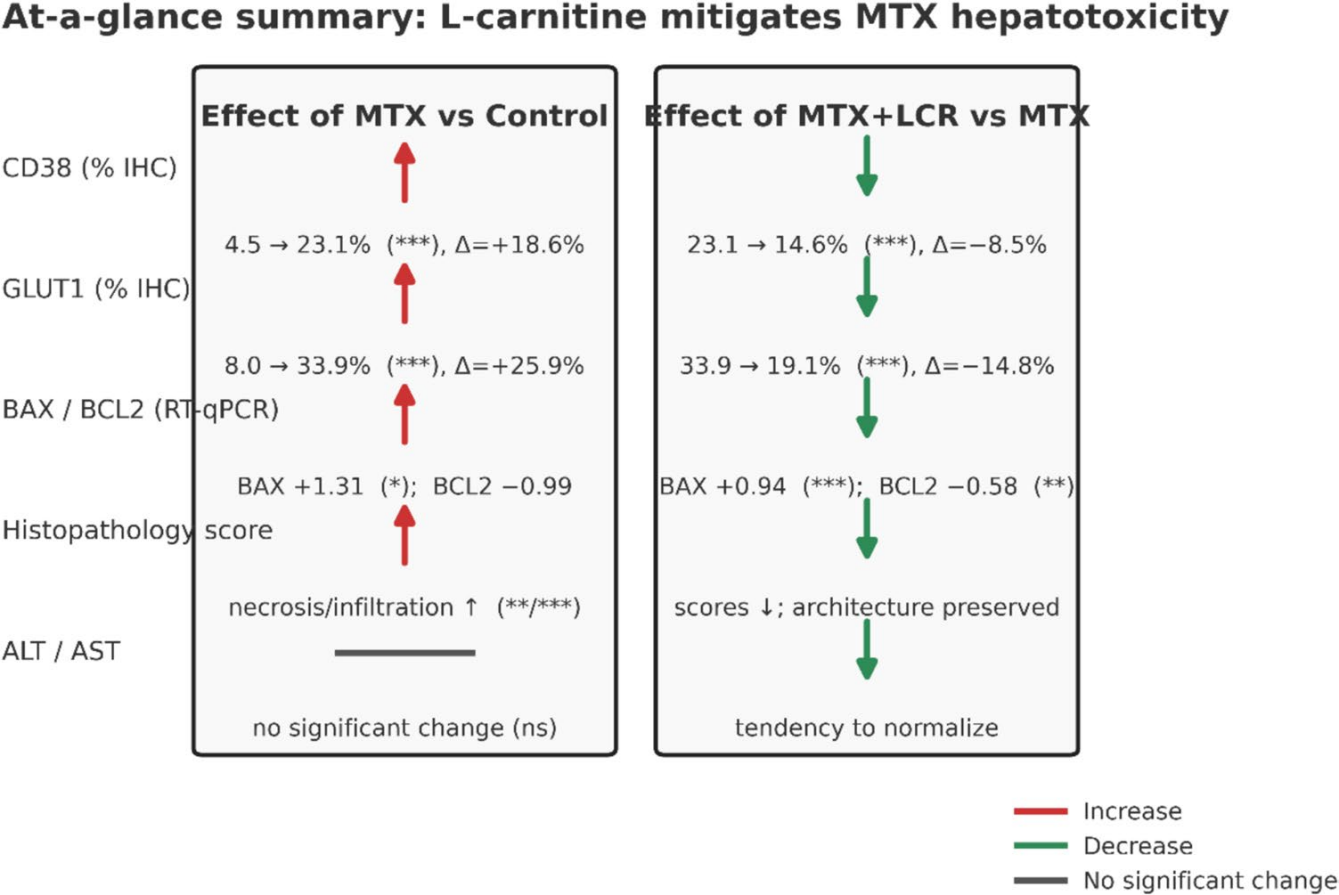
At-a-glance summary of the protective effects of L-carnitine against methotrexate (MTX)-induced hepatotoxicity. MTX markedly increased CD38 and GLUT1 expression, altered BAX/BCL2 balance, and aggravated histopathological scores, while L-carnitine co-administration significantly reduced these alterations, preserved liver architecture, and promoted normalization of molecular and histological parameters. ALT/AST showed no significant changes

As summarized in (Fig. 8), MTX increased CD38/GLUT1 and shifted the apoptotic balance toward cell death (BAX↑/BCL2↓) in parallel with worse histology; L-carnitine reduced CD38/GLUT1, partially normalized BAX/BCL2, and improved histology, while ALT/AST trended toward normalization.

### Study limitations and future perspectives

While the current results are promising, the study has several limitations. First, our design models acute, single-dose MTX exposure intended to capture early hepatotoxic events; however, clinical MTX regimens in humans are typically prolonged and cumulative. Evidence indicates that treatment duration and schedule can influence hepatic injury signatures and biomarker kinetics (Mansour et al. 2021; Abuzaid et al. 2025). To enhance translational relevance and methodological reproducibility, future studies should implement chronic/cumulative MTX protocols and longer follow-up windows. Second, this study was specifically designed as an acute hepatotoxicity model in non-tumor rats to capture early molecular and tissue-level events. Consequently, the potential impact of L-carnitine on tumor biology or the anticancer efficacy of MTX was beyond the scope of the present work. Nevertheless, future studies incorporating tumor-bearing models will be essential to determine whether L-carnitine can protect normal tissues without diminishing MTX’s chemotherapeutic potential.

## Conclusion

Our findings suggest that L-carnitine may exert hepatoprotective effects against MTX-induced hepatotoxicity in rats. These protective actions appear to be mediated through multiple complementary mechanisms, including:

Attenuation of oxidative stress, as evidenced by decreased expression of CD38 and GLUT1,

Modulation of apoptotic signaling, characterized by BAX suppression and BCL2 restoration,

Preservation of hepatic histoarchitecture, with reduced necrosis, inflammation, and hepatocellular degeneration and partial normalization of serum transaminases (ALT, AST), reflecting improved hepatocellular integrity.

By simultaneously influencing oxidative damage, inflammation, and apoptosis, L-carnitine may represent a potential adjuvant to mitigate MTX-associated hepatotoxicity. Further studies are warranted to evaluate its long-term efficacy, dose–response profiles, and potential synergistic interactions with other hepatoprotective agents.

## Data Availability

The datasets generated and/or analyzed during the current study are available from the corresponding author on reasonable request.

## Declarations

### Animal welfare statement

All experimental procedures were approved by the Burdur Mehmet Akif Ersoy University Animal Experiments Local Ethics Committee (Approval No: 1379, dated 02.10.2024). A total of 24 healthy male Wistar albino rats (250–300 g) were used, with six animals per group (n = 6). All procedures were conducted in accordance with Directive 2010/63/EU on the protection of animals used for scientific purposes. This study was conducted within the scope of the Süleyman Demirel University Scientific Research Projects Coordination Unit (BAP) project (No: 9524).
